# Longitudinal evaluation of discordant symmetric dimethylarginine and creatinine as markers of glomerular filtration rate in radioiodine-treated hyperthyroid cats

**DOI:** 10.1177/1098612X261466163

**Published:** 2026-06-26

**Authors:** Lorna Hardy, Angie Hibbert, Séverine Tasker, Natalie C Finch

**Affiliations:** 1 University of Bristol, Bristol, UK; 2 Bristol Vet Specialists, Bristol, UK; 3 The Feline Centre, Langford Vets, University of Bristol, Bristol, UK

**Keywords:** Kidney, chronic kidney disease, renal, biomarkers

## Abstract

**Objectives:**

The aim of the present study was to determine the frequency and clinical significance of discordant serum symmetric dimethylarginine (SDMA) and creatinine concentrations relative to glomerular filtration rate (GFR) in mature and senior hyperthyroid cats treated with radioactive iodine (RAI). Secondary aims of the study were to compare the sensitivity and specificity of two SDMA upper reference intervals (RIs; >14 and ⩾18 µg/dl) and creatinine (⩾175 µmol/l) for detecting decreased GFR and to evaluate the intra-individual variability of both biomarkers.

**Methods:**

A prospective longitudinal study of 27 client-owned hyperthyroid cats receiving low-dose RAI therapy was conducted. At baseline, 1, 6 and 12 months after RAI, cats were evaluated for thyroid status, GFR, SDMA and creatinine concentrations. Discordance was defined as one renal biomarker exceeding its RI with the other within/below the RI, using laboratory and age-specific SDMA RIs. In euthyroid cats, sensitivity, specificity and predictive values of SDMA/creatinine for decreased GFR and intra-individual variability were determined.

**Results:**

Discordance between SDMA and creatinine was in the range of 10–75% and was highest with the SDMA limit >14 µg/dl. SDMA-GFR discordance exceeded creatinine-GFR discordance at all time points and was highest at baseline, when all cats were hyperthyroid. In cats w﻿ith euthyroid status at 12 months, SDMA >14 µg/dl was more sensitive (83%) but less specific (50%) for decreased GFR than SDMA ≥18 µg/dl (sensitivity 50%, specificity 71%) and creatinine (sensitivity 50%, specificity 85%). Intra-individual variability was similar for both biomarkers (~15%).

**Conclusions and relevance:**

Discordant SDMA and creatinine concentrations are frequent in older hyperthyroid cats and are influenced by thyroid status and SDMA RIs, complicating interpretation. The SDMA limit ≥18 µg/dl improves specificity for decreased GFR and is more appropriate for senior cats. SDMA concentrations in the range of 15–18 µg/dl are less likely to reflect true GFR reduction when creatinine is normal; therefore, non-renal influences such as hyperthyroidism should be considered and ongoing monitoring of renal parameters is recommended.

## Plain language summary

A long-term assessment of two different indirect blood indicators of kidney function in cats undergoing radioiodine treatment for hyperthyroidism, to determine any discrepancies between the two and how these relate to direct measurements of kidney function

This study looked at how two common blood tests for kidney function, symmetric dimethylarginine (SDMA) and creatinine, behave in older cats treated with radioiodine for hyperthyroidism. Vets often use these tests to check how well the kidneys are functioning, but the two markers do not always agree. The researchers wanted to understand how often this happens and which test is more reliable when compared with a more direct measurement of kidney function. A total of 27 hyperthyroid cats were followed for 1 year. Before treatment with radioiodine and again at 1, 6 and 12 months afterwards, the cats had their thyroid levels, kidney glomerular filtration rate (GFR), SDMA and creatinine measured. The team looked for cases where one kidney marker (either SDMA or creatinine) was high but the other was normal or low, and they assessed how well each marker detected a true decrease in kidney filtration function by comparing them to GFR. The researchers found that SDMA and creatinine results did not agree in 10–75% of cats, especially when using a lower SDMA cut-off (>14 µg/dl). SDMA did not agree with the direct measure of kidney function, GFR, more frequently than creatinine, particularly when the cats were still hyperthyroid. At 12 months after treatment, in the cats with normal thyroid levels, the higher SDMA cut-off (⩾18 µg/dl) was more accurate and produced fewer false-positive results. Overall, the study shows that discrepancies in SDMA and creatinine results are common in older hyperthyroid cats, and thyroid disease itself can affect SDMA. SDMA values of 15–18 µg/dl may not truly indicate kidney disease if creatinine is normal. For senior cats, using the higher SDMA cut-off improves accuracy. When results do not match, vets should consider non-kidney factors, like thyroid status, and continue monitoring over time.

## Introduction

Chronic kidney disease (CKD) and hyperthyroidism are common comorbidities in mature (7–10 years) and senior (>10 years) cats.^1–3^ Restoration of euthyroidism after treatment of hyperthyroidism can result in unmasking decreased renal filtration function associated with CKD as the hyperthyroid state increases glomerular filtration rate (GFR).^4^ Reduced renal filtration function is most sensitively detected by decreased GFR, which has been reported in 29% of hyperthyroid cats in the 12 months after radioactive iodine (RAI) treatment;^5^ therefore monitoring of renal function is recommended.^4,5^ In the clinical setting, direct measurement of GFR is rarely performed because of the requirement for multiple samples to be collected over several hours, costs of analysis and lack of standardisation in interpretation. Therefore, routine evaluation of renal filtration function is indirectly measured using serum concentrations of the renal biomarkers symmetric dimethylarginine (SDMA) and creatinine.^6,7^ Detection of persistent increased serum concentrations of these biomarkers, in the absence of any prerenal or postrenal causes, is fundamental for the diagnosis and staging of CKD as outlined by the International Renal Interest Society (IRIS).^7^

The reference interval (RI) for the commercially available immunoassay for the measurement of serum SDMA classifies serum SDMA >14 µg/dl as increased and consistent with decreased GFR.^8^ However, a recent study investigating age-specific RIs in healthy mature and senior cats determined that an upper reference limit of ≥18 µg/dl for serum SDMA concentrations may be more appropriate and that the upper limit of >14 µg/dl may overestimate renal dysfunction.^9^ The suggested age-specific RI of ≥18 µg/dl is also in line with the SDMA concentrations stated in the IRIS recommendations for staging of IRIS stage 2 CKD in cats.^7^ Therefore, in the older population of cats that develop hyperthyroidism, applying the upper reference limit of ≥18 µg/dl may be appropriate for monitoring renal function.

Hyperthyroidism can affect biomarkers of renal filtration function due to glomerular hyperfiltration, and loss of lean muscle mass in hyperthyroid cats may decrease endogenous creatinine production and thus circulating creatinine concentrations.^10^ A physiological influence of hyperthyroidism on SDMA metabolism is also possible, and previous studies suggest that thyroid status likely influences SDMA concentrations in cats.^11,12^ In human patients with hyperthyroidism, increased SDMA concentrations have been reported in the absence of concurrent renal disease.^13,14^ These findings suggest that production or metabolism of SDMA may be altered by thyroid dysfunction independent of altered GFR.

In the clinical setting, when investigating potential decreased renal function, both SDMA and creatinine are frequently available in biochemistry panels. There have been few studies performed on the clinical application of SDMA to guide decision-making.^15^ Therefore, in the clinical scenario whereby SDMA and creatinine results are discordant (ie, one is increased above the RI while the other is not), the clinician faces a conundrum in the interpretation of GFR. The primary aim of this study was to describe the frequency of discordant SDMA and creatinine concentrations in relation to GFR, utilising both the laboratory RI greater than 14 µg/dl and the proposed age-specific RI (⩾18 µg/dl)^9^ for SDMA, over a 12-month follow-up period in a population of mature and senior hyperthyroid cats treated with RAI and to determine the effect of hyperthyroidism on discordance. Secondary aims of the study were to determine the sensitivity, specificity and predictive values of both upper limits of SDMA (>14 and ⩾18 µg/dl) and creatinine in detecting decreased GFR and the intra-individual variation of markers in euthyroid cats.

## Materials and methods

### Study population

A total of 27 client-owned hyperthyroid cats presenting to a referral hospital for low-dose (3 mCi; 111 MBq) RAI treatment had been recruited prospectively into a study and the cohort followed longitudinally for a 12-month follow-up period; thyroid function (total thyroxine [TT4] and thyroid stimulating hormone [TSH]), serum creatinine concentration and GFR data from these cats have been published previously.^5^ Cats had been evaluated at the time of recruitment into the study for baseline measurements (T0) and at 1- (T1), 6- (T2) and 12-month (T3) time points after RAI treatment. Each assessment comprised a full physical examination, systolic blood pressure via Doppler technique, and blood and urine sampling. The study was approved by the University of Bristol Animal Welfare and Ethics Review Body (VIN/12/025).

### Measurement of thyroid status

Serum TT4 had been measured using a chemiluminescent immunoassay at a commercial reference laboratory (Langford Diagnostic Laboratories, Langford Vets, University of Bristol, UK)^16^ while serum TSH was measured using a validated canine assay at a commercial reference laboratory (Royal Veterinary College Diagnostic Laboratory Services, Hatfield, UK).^17^

Each cat was classified into one of four possible thyroid statuses at each of the four time points:

- Hypothyroid: TT4 below 15 nmol/l (<1.2 µg/dl), TSH above 0.3 ng/ml
- Euthyroid: TT4 less than or equal to 60 nmol/l (⩽4.7 µg/dl), TSH less than or equal to ⩽0.3 ng/ml
- Subclinical hypothyroid: TT4 15–60 nmol/l (1.2–4.7 µg/dl), TSH above 0.3 ng/ml
- Hyperthyroid: TT4 above 60 nmol/l (>4.7 µg/dl), TSH below 0.03 ng/ml

This classification resulted in 4/27 cats being reclassified from the statuses published in the original study by Finch et al^5^ due to revision of TSH concentrations for classifications of hypothyroidism.^5,18^

### Measurement of renal filtration function

Glomerular filtration rate was determined using a slope-intercept iohexol clearance method described by Finch et al,^19^ and GFR was defined as being decreased if it was below 0.92 ml/min/kg.^20^ Serum creatinine concentration was measured at Langford Diagnostic Laboratories, with increased creatinine concentration defined as 175 µmol/l or higher (⩾2.0 mg/dl), the upper limit of the laboratory’s RI.

Measurement of SDMA was performed on serum samples that had been stored at −80°C before analysis. SDMA analysis was performed approximately 16 months after data collection was completed for the original study. SDMA was determined using a validated immunoassay IDEXX SDMA Test (IDEXX Laboratories) with increased SDMA concentration defined as >14 µg/dl. An age-specific RI of ≥18 µg/dl was also evaluated.^9^

### Data analysis

Data analysis consisted of descriptive statistics.

#### Discordant SDMA and creatinine concentrations in relation to GFR

Discordant SDMA and creatinine was defined as being when either of these biomarkers was above the laboratory RI (>14 µg/dl and ⩾175 μmol/l, respectively) while the other was within or below the RI. Discordance was also determined by applying the age-specific RI of ≥18 µg/dl for SDMA. In cats with discordant SDMA and creatinine concentrations, the discordance between SDMA and GFR, and creatinine and GFR was subsequently determined. Discordance with GFR was defined as being when either GFR was below 0.92 ml/min/kg and either creatinine or SDMA were within or below the laboratory RI, or GFR was normal (⩾0.92 ml/min/kg) and either biomarker was above the upper limit of the laboratory’s RI.

#### Sensitivity, specificity, and predictive values of serum creatinine and SDMA concentrations in detecting decreased GFR in euthyroid cats

The sensitivity, specificity, negative predictive value (NPV) and positive predictive value (PPV) for the two upper limits of SDMA and creatinine for detecting decreased GFR were determined at the 12-month time point (T3). This time point was selected as it had the greatest proportion of cats with euthyroid status and cats with decreased GFR to include in the analysis. True positive was defined as decreased GFR with increased biomarker (SDMA or creatinine) serum concentrations, false positive as a biomarker above the laboratory RI with normal GFR, true negative as normal GFR with biomarker concentrations (SDMA or creatinine) within the RI and false negative as a biomarker within or below the laboratory RI with decreased GFR. The standard formulae included below were used to determine sensitivity, specificity, NPV and PPV.

$$\begin{array}{l} Sensitivity=true\;positive/\\ \left(true\;positive+false\;negative\right) \end{array}$$

$$\begin{array}{l} \mathrm{Specificity}=\mathrm{true}\mathrm{negative}/\\ \left(\mathrm{true}\mathrm{negative}+\mathrm{false}\mathrm{positive}\right) \end{array}$$

NPV=truenegative/(truenegative+falsenegative)

PPV=truepositive/(truepositive+falsepositive)

#### Intra-individual variability in longitudinal measurements of serum SDMA and creatinine in euthyroid cats

The intra-individual variability in longitudinal measurements of serum SDMA and creatinine was evaluated by determining the coefficient of variation percentage, calculated by (SD/mean) × 100. At least two SDMA or creatinine measurements were required at time points when cats had euthyroid status.

## Results

**Figure 1:**
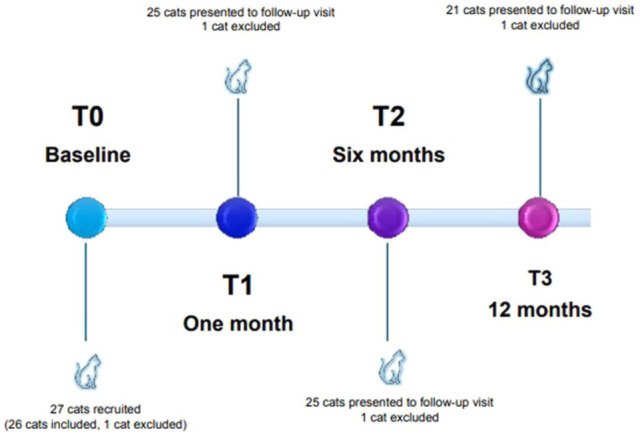
Study timeline outlining the number of cats analysed at each time point (baseline [T0], 1 [T1], 6 [T2] and 12 months [T3] after radioactive iodine treatment)

**Figure 2:**
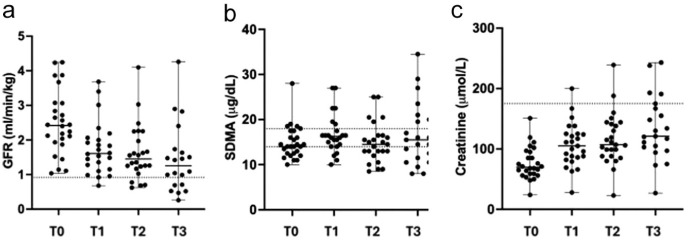
Measurements of renal filtration function in cats at baseline (T0), 1 (T1), 6 (T2) and 12 months (T3) after radioactive iodine treatment for (a) glomerular filtration rate (GFR), (b) serum symmetric dimethylarginine (SDMA) and (c) serum creatinine. The middle solid line represents the median value and the solid lines above and below this represent the range. The dashed line indicates the value below which GFR was defined as decreased renal filtration function and the upper limit of the reference intervals for serum SDMA and creatinine concentration

A total of 27 cats had been recruited to the study; 25 completed the 1- (T1) and 6-month (T2) follow-up visit and 21 completed the 12-month (T3) time points. The median age at recruitment was 11 years (range 7–16). Of the 27 cats, 15 were female neutered, 11 were male neutered and one was female entire. Median (range) TT4, TSH and creatinine concentrations, and GFR have been published previously.^5^ Serum creatinine concentrations had not been measured in one cat at T0 and SDMA had not been measured in one cat at the T1 time point due to insufficient sample volume. GFR measurement was also not available in one cat at the T2 time point and in one cat at the T3 time point. These four cats with incomplete data sets were excluded from analysis at the respective timepoints, leaving 26, 24, 24 and 20 cats for analysis (Figure 1). A summary of thyroid function status at each time point is provided in Table 1. The median (range) GFR, serum creatinine and SDMA concentrations are presented in Figure 2.

**Table 1:** Thyroid status of cats at baseline (T0), 1 (T1), 6 (T2) and 12 months (T3) after radioactive iodine therapy

Time point	T0 (n = 26)	T1 (n = 24)	T2 (n = 24)	T3 (n = 20)
Hyperthyroid	26 (100)	7 (29)	2 (8)	2 (10)
Euthyroid	0 (0)	16 (67)	18 (75)	16 (80)
Subclinical hypothyroid	0 (0)	0 (0)	1 (4)	0 (0)
Hypothyroid	0 (0)	1 (4)	3 (13)	2 (10)

Data are n (%)

### Discordant SDMA and creatinine concentrations in relation to GFR

**Figure 3:**
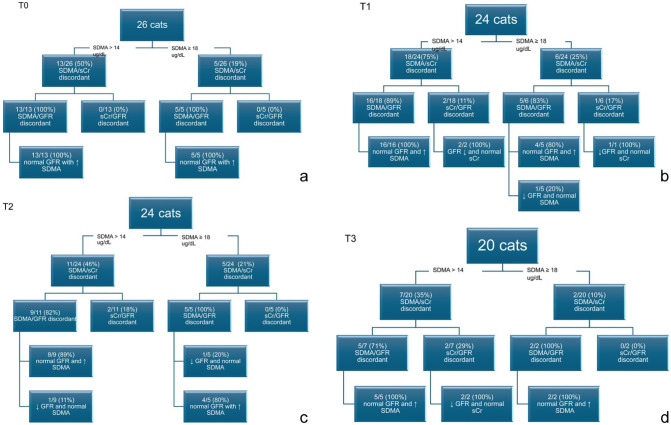
(a–d) Discordant symmetric dimethylarginine (SDMA) and serum creatinine (Cr) results in cats at (a) baseline (T0), (b) 1 (T1), (c) 6 (T2) and (d) 12 months (T3) after radioactive iodine treatment for both laboratory (>14 µg/dl) and age-specific (⩾18 µg/dl) SDMA reference intervals. Of those cats with discordant SDMA and Cr results, SDMA-glomerular filtration rate (GFR) and creatinine-GFR discordance is then presented below at each time point

The proportion of discordant results at each time point using both laboratory (>14 µg/dl) and age-specific (⩾18 µg/dl) SDMA RIs are presented in Figure 3a–d.

At T0, all cats were hyperthyroid and none of the cats had decreased GFR or increased serum creatinine. Using the SDMA upper reference limit of >14 µg/dl, 13/26 (50%) cats had discordant SDMA and creatinine concentrations (Figure 3a). Of these 13 cats, SDMA and GFR were discordant in all cats (100%). Using the alternative upper reference limit of ≥18 µg/dl, 5/26 (19%) cats had discordant SDMA and creatinine concentrations, of which 100% had discordant SDMA and GFR. At T1, T2 and T3, 18/24 (75%), 11/24 (46%) and 7/20 (35%) cats, respectively, had discordant SDMA and creatinine concentrations when the upper reference limit of >14 µg/dl and above was applied. Discordance of SDMA and GFR was higher than that of creatinine and GFR at all time points (SDMA vs GFR at T1, T2 and T3 was 16/18 (89%), 9/11 (82%) and 5/7 (71%), respectively, and creatinine vs GFR at T1, T2 and T3 was 2/18 (11%), 2/11 (18%) and 2/7 (29%), respectively) (Figure 3b–d).

When the upper reference limit of ≥18 µg/dl was used for SDMA, at T1, T2 and T3, 6/24 (25%), 5/24 (21%) and 2/20 (10%) cats, respectively, had discordant SDMA and creatinine concentrations (Figure 3b–d). Discordance of SDMA and GFR was higher than that of creatinine and GFR at all time points (SDMA vs GFR at T1, T2 and T3 was 5/6 (83%), 5/5 (100%) and 2/2 (100%), respectively, and creatinine vs GFR at T1, T2 and T3 was 1/6 (17%), 0/5 (0%) and 0/2 (0%), respectively) (Figure 3b–d).

Concordant results are presented in S1 in the supplementary material.

### Sensitivity, specificity and predictive values of serum creatinine and SDMA concentrations in detecting decreased GFR at T3

A total of 17 cats were euthyroid at the T3 time point, of which 16 had complete data available for analysis. Of the euthyroid cats, 6/16 (38%) developed decreased GFR at T3. The sensitivity, specificity, NPV and PPV for detecting decreased GFR was 83%, 50%, 88% and 42% for SDMA (>14 µg/dl), 50%, 71%, 77% and 43% for SDMA (⩾18 µg/dl), and 50%, 85%, 80% and 60% for creatinine, respectively (Table 2).

**Table 2:** Sensitivity, specificity, negative predictive value (NPV) and positive predictive value (PPV) of increased creatinine (>175 umol/l) and symmetric dimethylarginine (SDMA) (>14 µg/dl and ≥18 µg/dl) in detecting reduced glomerular filtration rate in cats with euthyroid status at 12 months (T3) after radioactive iodine therapy

	Sensitivity	Specificity	NPV	PPV
SDMA >14 µg/dl	83	50	88	42
SDMA ⩾18 µg/dl	50	71	77	43
Creatinine	50	85	80	60

Data are %

### Intra-individual variability in longitudinal measurements of serum SDMA and creatinine

**Figure 4:**
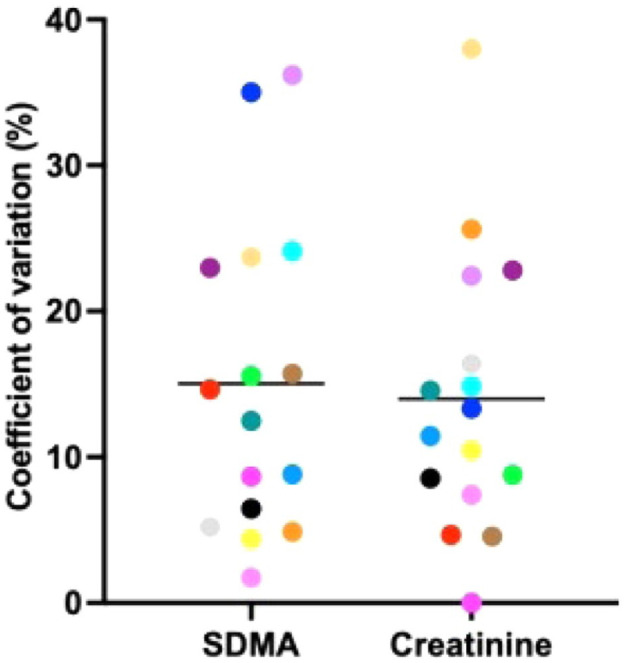
Coefficient of variation of longitudinal measurements of symmetric dimethylarginine (SDMA) and creatinine in 16 euthyroid cats. The bold line represents the mean. Each individual cat is represented by the same colour circle in the SDMA and creatinine data

The intra-individual variability in longitudinal measurements of serum SDMA and creatinine were evaluated in 16 cats at euthyroid status time points and was similar. The mean coefficient of variation for the 16 cats for SDMA was 15% and for creatinine was 14%. Individual coefficients of variation for each of the 16 cats for SDMA and creatinine are presented in Figure 4.

## Discussion

The present study identified a high frequency of discordance (10–75% of cats) between creatinine and SDMA concentrations at all time points in this study population of mature and senior cats in hyperthyroid and euthyroid states. There was greater discordance identified when the laboratory upper reference limit of >14 µg/dl was applied compared with the proposed age-specific reference limit of ≥18 µg/dl. The highest number of discordant SDMA/creatinine results was observed at T1 and, of the discordant cats, 6/18 (33%) were hyperthyroid. Discordance rates between SDMA and GFR were higher at all time points compared with those of creatinine and GFR. There was 100% discordance between SDMA and GFR vs 0% discordance between creatinine and GFR at the baseline (T0) time point, when 100% of the cats were hyperthyroid, when applying both upper limits of SDMA, suggesting that hyperthyroidism is an important non-renal factor influencing SDMA in cats.

The SDMA upper limit of >14 µg/dl was more sensitive at detecting decreased GFR compared with the SDMA upper limit of ≥18 µg/dl and creatinine. However, the upper limit of >14 µg/dl for SDMA was less specific (50%) than ≥18 µg/dl (71%) and serum creatinine (85%) for the detection of decreased GFR. There are a limited number of studies that have analysed the diagnostic accuracy of SDMA in cats, and reported sensitivities and specificities have varied greatly (between 15%^21^ and 100%^22^ and between 75%^23^ and 98%,^24^ respectively). Initial studies reported SDMA to be a more sensitive marker for decreased renal function when compared with creatinine, with increases in SDMA concentration first detected 17 months before increases in creatinine concentration in cats developing CKD.^22^ However, subsequent studies have reported that the relationship between SDMA and GFR and between creatinine and GFR are comparable.^23,25^ Comparison of these studies is limited by the variability in study design, population analysed and the reference standard for which they determined impaired renal filtration function.^15^ Unlike creatinine, SDMA is not considered to be affected by muscle mass or sex and may be more reliable in inflammatory states.^26,27^ However, evidence from studies suggest it may be influenced by non-renal factors, including lymphoma^28,29^ and diabetes mellitus.^30,31^

Importantly, the findings of the present study suggest SDMA may be influenced by hyperthyroidism, which may decrease its specificity in detecting decreased GFR relative to creatinine. When facing the clinical dilemma of discordance between SDMA and creatinine in cats, non-renal factors, such as hyperthyroidism, should be excluded. In addition, ongoing monitoring of renal function, including serial measurement of renal biomarkers and urinalysis, should be performed, as increased SDMA may suggest subsequent development of decreased GFR. Intra-individual variability of both biomarkers was similar, and therefore increases in both biomarkers of greater than 15% may be of clinical significance in euthyroid cats.

In a previous study of hyperthyroid cats undergoing bilateral thyroidectomy, 35% of samples had discordant SDMA and creatinine concentrations, with SDMA most often increased while creatinine concentration remained within the RI.^11^ However, the biomarkers were not measured against GFR for comparison. A further study found a lack of correlation between SDMA and creatinine in hyperthyroid cats before RAI treatment but a positive correlation after RAI treatment, with the authors speculating that this was due to the hyperthyroidism-induced metabolic state.^12^ SDMA is positively correlated with free T4 in humans and can be increased in humans with hyperthyroidism.^14,32^ Potential mechanisms for this included genetic variation, increased synthesis of arginine N-methyltransferase involved in L-arginine methylation, or decreased SDMA metabolism due to deceased enzymatic hydrolysis.^14,33^ It is clear that SDMA is increased in both cats and humans with hyperthyroidism, despite higher GFR, which should increase renal clearance of SDMA. Whether increased SDMA concentrations in cats with hyperthyroidism is of pathophysiological significance is unclear. Previous studies of hyperthyroid cats have shown that increased SDMA predicts declining renal function.^21,24^ Although such studies can only demonstrate associations, and not causality, one can postulate that SDMA may not only be a predictor of declining renal function but also a mediator. SDMA is a competitive inhibitor of arginine transport and so decreases endothelial nitric oxide (NO) synthesis, probably by limiting L-arginine supply to endothelial NO synthase, and in this way SDMA may play a role in vascular endothelial dysfunction. Indeed, SDMA decreased NO synthesis and increased reactive oxygen species in cultured endothelial cells in a dose-dependent manner.^34^

The present study identified higher sensitivity of SDMA >14 µg/dl for the detection of decreased GFR than SDMA of ≥18 µg/dl and creatinine in the euthyroid population (83% vs 50% vs 50%), similar to previously reported.^22^ Serum SDMA >14 µg/dl was less specific (50%) for detecting decreased GFR than both the SDMA upper limit of ≥18 µg/dl and serum creatinine (71% and 85%, respectively), which is considerably lower than the 91% specificity reported by Hall et al.^22^ Higher sensitivity of SDMA >14 µg/dl, compared with creatinine, for predicting azotaemia in euthyroid cats after RAI,^24^ and lower specificity of SDMA >14 µg/dl, compared with creatinine, in detecting decreased renal function post-RAI oral therapy,^35^ have been reported previously.^24,35^ The present study also identified lower PPVs for both SDMA >14 µg/dl and creatinine (42% and 60%, respectively), compared with a previous study (86% and 100%, respectively).^22^ This may be partly influenced by the lower prevalence of decreased GFR (31%) in our population and smaller population size compared with the previous study and the different upper limit of serum creatinine that was used (≥175 μmol/l in this study compared with 140 μmol/l).^22^ However, we consider our study population to be representative of the older feline population in which SDMA and creatinine concentrations would be routinely measured, and therefore consider our findings relevant.

This is the first study reporting the intra-individual biological variability in SDMA over a long (6–12 months) follow-up period in mature and senior cats. The intra-individual variability in SDMA and creatinine was evaluated to understand if this was a potential contributing factor to discordance. We found that variability, as determined by the mean coefficient of variation, was similar in SDMA and creatinine measurements (15% vs 14%) in euthyroid cats. Van Vertloo et al^12^ observed variability in SDMA concentrations in individual cats across time points, although the coefficient of variation as an objective measurement was not determined. A study evaluating the analytical performance of SDMA in healthy cats aged 1–7 years over a 6-week period reported intra-individual variability to be approximately 19.9%.^36^ The variability in creatinine concentration was higher in the present study than that reported in a previous study of euthyroid cats (14% vs 8.5%).^37^ This may reflect the longer follow-up period for repeated measurements in the present study (6–12 months) compared with the previous study (6 months only). Breed can also influence variation, with Birman cats reported to have physiologically higher SDMA and creatinine compared with other breeds.^38^ No Birman cats were included in this study cohort.

A previous study defined progressive CKD as an increase in plasma creatinine concentration of at least 25% relative to the concentration at diagnosis;^39^ the variability of the individual creatinine results in the present study (14%) may suggest that progression (ie, decrease in GFR) may be associated with a lower relative increase in creatinine concentration.^39^ However, analytical variation associated with analyser imprecision should be considered as this will also contribute to variation between replicate measurements.^39,40^ A previous study identified a lower intra-individual variability and reference change value for creatinine compared with SDMA (enzyme multiplied immunoassay technique) (8.6% vs 19.9% and 24.7% vs 61.7%, respectively), suggesting that more variability can occur in repeated SDMA measurements in an individual cat that may not be clinically significant.^41^ Previous studies looking at the dispersion of measured serum SDMA, which describes the range of possible values for a test result based on individual biological and analyser variation, identified a wider dispersion for SDMA compared with creatinine.^36,40,42^

Limitations of the present study include the small sample size. The gold standard method of liquid chromatography mass spectrometry was not utilised for SDMA measurement; however, the IDEXX laboratory test used is validated and therefore was considered appropriate and most representative of the methodology used for samples submitted from clinical practice.^43^ The SDMA and creatinine measurements in this study were analysed at different diagnostic laboratories, but this may also reflect the situation that can occur for samples submitted from clinical practice. In addition, it is possible that muscle mass may have influenced the serum creatinine results, which was not assessed in this study.

## Conclusions

There was a high frequency of discordance between serum SDMA and creatinine, and SDMA and GFR, particularly when cats were hyperthyroid. The discordance was lower when the upper RI of ≥18 µg/dl was applied, indicating that the proposed age-specific RI may be more appropriate for the older cat population. The upper limit of ≥18 µg/dl for SDMA concentrations was more specific for detecting decreased GFR than when applying the upper limit of >14 µg/dl. However, serum SDMA >14 µg/dl was more sensitive for detecting decreased GFR than ≥18 µg/dl, highlighting that cats with SDMA >14 µg/dl may require ongoing monitoring of their renal parameters, including serial measurement of renal biomarkers and urinalysis. The results of the present study suggest that when faced with the clinical dilemma of discordant SDMA and creatinine results in mature and senior cats, a serum SDMA of ≥18 µg/dl should be considered more significant for the detection of decreased GFR, whereas an SDMA concentration of 15–18 µg/dl has a lower likelihood of indicating a true reduction in GFR, particularly if creatinine is within the RI. Furthermore, we should consider that an increase in SDMA may be influenced by non-renal factors, such as hyperthyroidism.

## Supplemental Material

S1a–d: Concordant symmetric dimethylarginine (SDMA) and serum creatinine (sCr) results in cats at baseline (T0), 1 (T1), 6 (T2) and 12 months (T3) after radioactive iodine treatment for both laboratory (>14 μg/dl) and age-specific (≥18 μg/dl) SDMA reference intervals. Of those cats with concordant SDMA and creatinine results, SDMA/sCr and glomerular filtration rate (GFR) concordance and SDMA/sCr and GFR concordance is then presented below at each time point.
