# Supplementary material for: Longitudinal evaluation of discordant symmetric dimethylarginine and creatinine as markers of glomerular filtration rate in radioiodine-treated hyperthyroid cats
Source: J Feline Med Surg. 2026 Jun 26;28(8):1098612X261466163. doi: 10.1177/1098612X261466163 (PMC13482059; doi:10.1177/1098612X261466163)
Supplement: Supplementary file 1 [file sj-pdf-1-jfm-10.1177_1098612X261466163.pdf]

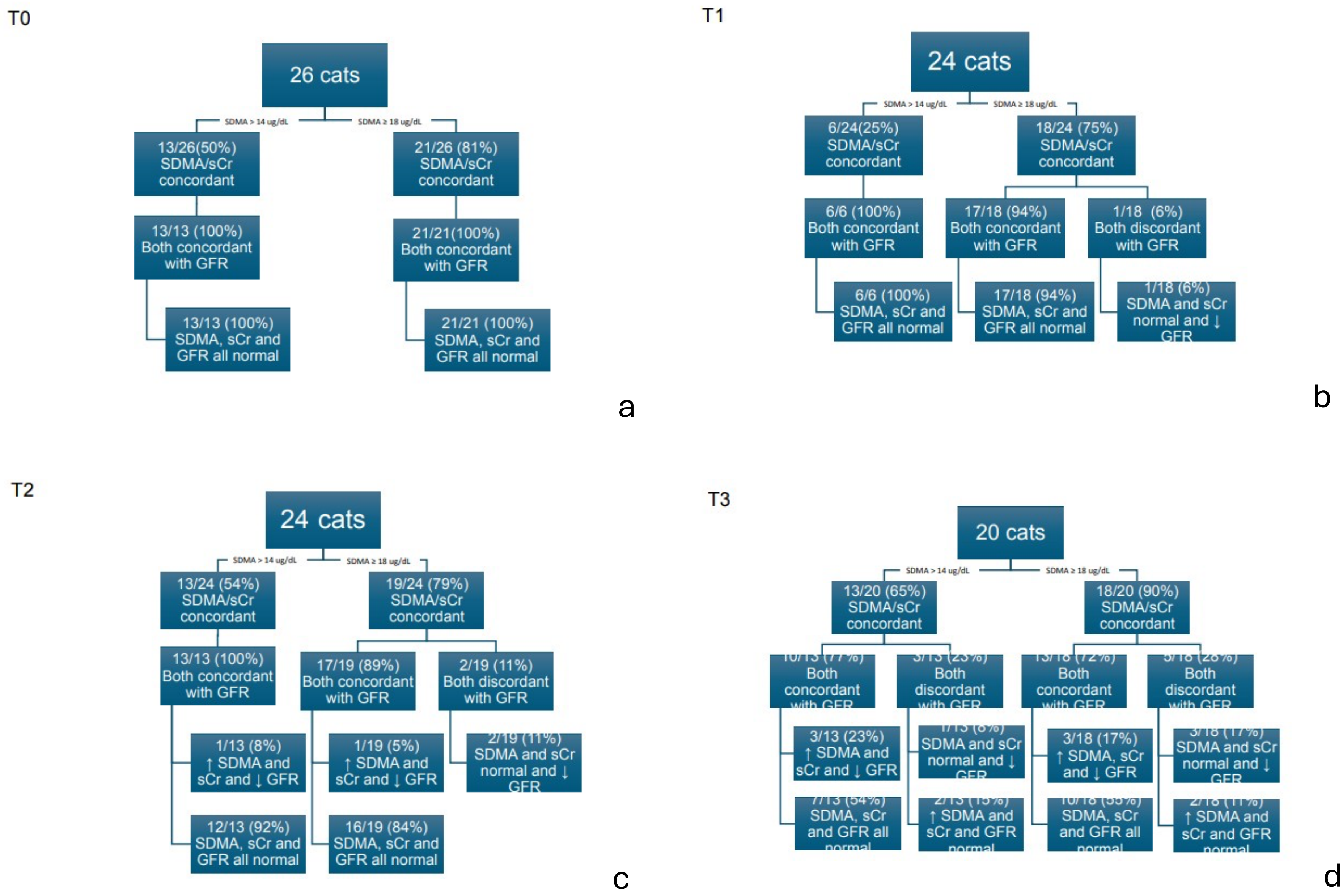
T0
26 cats
SDMA > 14 ug/dL
SDMA ≥ 18 ug/dL
13/26(50%) SDMA/sCr concordant
21/26 (81%) SDMA/sCr concordant
13/13 (100%) Both concordant with GFR
21/21(100%) Both concordant with GFR
13/13 (100%) SDMA, sCr and GFR all normal
21/21 (100%) SDMA, sCr and GFR all normal
a
T1
24 cats
SDMA > 14 ug/dL
SDMA ≥ 18 ug/dL
6/24(25%) SDMA/sCr concordant
18/24 (75%) SDMA/sCr concordant
6/6 (100%) Both concordant with GFR
17/18 (94%) Both concordant with GFR
1/18 (6%) Both discordant with GFR
6/6 (100%) SDMA, sCr and GFR all normal
17/18 (94%) SDMA, sCr and GFR all normal
1/18 (6%) SDMA and sCr normal and ↓ GFR
b
T2
24 cats
SDMA > 14 ug/dL
SDMA ≥ 18 ug/dL
13/24 (54%) SDMA/sCr concordant
19/24 (79%) SDMA/sCr concordant
13/13 (100%) Both concordant with GFR
17/19 (89%) Both concordant with GFR
2/19 (11%) Both discordant with GFR
1/13 (8%) ↑ SDMA and sCr and ↓ GFR
1/19 (5%) ↑ SDMA and sCr and ↓ GFR
2/19 (11%) SDMA and sCr normal and ↓ GFR
12/13 (92%) SDMA, sCr and GFR all normal
16/19 (84%) SDMA, sCr and GFR all normal
c
T3
20 cats
SDMA > 14 ug/dL
SDMA ≥ 18 ug/dL
13/20 (65%) SDMA/sCr concordant
18/20 (90%) SDMA/sCr concordant
10/13 (77%) Both concordant with GFR
3/13 (23%) Both discordant with GFR
13/18 (72%) Both concordant with GFR
5/18 (28%) Both discordant with GFR
3/13 (23%) ↑ SDMA and sCr and ↓ GFR
1/13 (8%) SDMA and sCr normal and ↓ GFR
3/18 (17%) ↑ SDMA, sCr and ↓ GFR
3/18 (17%) SDMA and sCr normal and ↓ GFR
7/13 (54%) SDMA, sCr and GFR all normal
2/13 (15%) ↑ SDMA and sCr and GFR normal
10/18 (55%) SDMA, sCr and GFR all normal
2/18 (11%) ↑ SDMA and sCr and GFR normal
d
